# Dietary intake of n-3 PUFAs modifies the absorption, distribution and bioavailability of fatty acids in the mouse gastrointestinal tract

**DOI:** 10.1186/s12944-016-0399-9

**Published:** 2017-01-17

**Authors:** Qin Yang, Shunhe Wang, Yunqi Ji, Haiqin Chen, Hao Zhang, Wei Chen, Zhennan Gu, Yong Q. Chen

**Affiliations:** 1 State Key Laboratory of Food Science and Technology, School of Food Science and Technology, Jiangnan University, 1800 Lihu Avenue, Wuxi, 214122 People’s Republic of China; 2 Synergistic Innovation Center for Food Safety and Nutrition, Wuxi, 214122 People’s Republic of China; 3 Department of Cancer Biology and Biochemistry, Wake Forest School of Medicine, Winston-Salem, NC 27157 USA

**Keywords:** n-3 PUFA, SFA, Gut, Distribution, Bioavailability, Absorption

## Abstract

**Background:**

Dietary polyunsaturated fatty acids (PUFAs), especially n-3 PUFAs, are important for human health. The intestinal tract, a location that is heavily colonized by microorganisms, is the main organ for absorbing fatty acids.

**Methods:**

The purpose of this study was to analyze the effects of dietary n-3 and n-6 PUFAs on the distribution of different types of fatty acids and their bioavailability along the gut. Mice were fed for a week with experimental diets containing high n-3 or high n-6 fatty acid levels. Blood was collected at different time points, and after 7 days the mice were euthanized and their digestive tract was divided into 17 segments for fatty acids analyses.

**Results:**

We found that supplementing n-3 fatty acids significantly changed the ratio of n-6/n-3 PUFAs, increased the bioavailability of n-3 PUFAs, and altered fatty acid distribution. In addition, in the n-3 diet group, the absorption of saturated fatty acids (SFAs) along the gut was found to be inhibited, which was confirmed by feeding the mice with a diet containing deuterium-labeled palmitic acid and stearic acid.

**Conclusion:**

These results show that a diet rich in n-3 PUFAs can significantly modify the distribution and bioavailability of fatty acids, and particularly, may block the absorption of SFAs in the mouse gastrointestinal (GI) tract.

**Electronic supplementary material:**

The online version of this article (doi:10.1186/s12944-016-0399-9) contains supplementary material, which is available to authorized users.

## Background

Dietary lipids are important for human health, providing not only caloric energy, but also taking part in vital cellular functions. High fat consumption and high ratios of n-6/n-3 polyunsaturated fatty acids (PUFAs) in the Western diet are associated with an increased incidence of obesity, diabetes, cardiovascular disease and cancer [1, 2]. While saturated fatty acids (SFAs) and monounsaturated fatty acids (MUFAs) can be synthesized de novo, PUFAs must be obtained via dietary intake. A low ratio of n-6/n-3 PUFAs may be more important than the total amount of n-3 PUFAs for health benefits [3, 4].

Different dietary fatty acids are incorporated into the body with different patterns and efficiencies [5]. Many factors contribute to this difference in lipid response to the diet. While the genetic background influences dietary lipid absorption and metabolism [6], evidence suggest that the gut microenvironment, including its complicated environment and the gut microbiota, plays an important role in fat digestion, absorption and metabolism [7, 8]. Fatty acids are distributed unevenly along the gut. The bioavailability of fatty acids is associated with gastrointestinal function, and alterations in their metabolism may cause mucosal inflammation and other gastrointestinal troubles [9]. Supplementation of n-3 PUFAs can improve gut function via interaction with the gut microbiota [10].

In this study, we focused on the effects of high and low ratios of n-3 PUFAs on the distribution of fatty acids, including SFAs, MUFAs and PUFAs, and analyzed in detail the resulting modifications of the uptake of n-3 PUFAs along the gut.

## Methods

### Animals and diets

Six-week-old male C57BL/6 J mice were obtained from the Shanghai Laboratory Animal Center (Shanghai, China). After 1 week of acclimatization with free access to standard mouse chow (Commercial diet, 17.14% of energy from fat, 5.05 g/100 g) and water, the mice were randomly divided into three groups (10 mice were used in a group) and fed a high n-3 PUFA diet (n-6:n-3 ratio = 1:1), a high n-6 PUFA diet (n-6:n-3 ratio = 40:1) or control diet (n-6:n-3 ratio = 20:1) for a week. Diets were prepared by the custom animal diet laboratory of the Animal Resources Program at Jiangnan University (Additional file 1: Table S1). All 3 diets contained 397 kcal/100 g, 13 g fat/100 g, and 29.2% of energy was from fat, 46.4% from carbohydrates, and 24.4% from proteins.

Two deuterium labeled stable isotopes, hexadecanoic-*d*_*31*_ acid (*d*_31_-C16:0, 98.9 atom% D, D-2002, lot No. M-363) and octadecanoic-*d*_*35*_ acid (*d*_35_-C18:0, 99.2 atom% D, D-2007, lot No. M-413-A) which were purchased from C/D/N Isotopes Inc. (Poínte-Claíre, Quebec, Canada), were added to experimental diets at a concentration of 0.14 g/100 g dry weight diet (Additional file 1: Table S1) and fed a new cohort of mice which were also divided into control, n-6 PUFA and n-3 PUFA diet groups (5 mice in each group).

All animals were maintained in an isolated environment in barrier cages and fed the assigned special diet restricted to 10 g/mouse/day. Daily intake of diet of each cage of mice were recorded and the average dietary intake of a mouse in 24 h was calculated accordingly, for details please reference Additional file 1: Table S2. Animal care and protocols were approved by the Jiangnan University Animal welfare and Ethics Committee.

### Blood and tissue collection

The C57BL/6 J mice were fed their respective experimental diet for 1 h, 3 h, 6 h, 12 h, 24 h, 3 days, and 7 days, at which times approximately 50 μL blood was collected through the retro-orbital vein, snap-frozen in liquid nitrogen, and stored at −80 °C until analysis. To avoid overdraw of blood from the mice, each group was divided into two subgroups. Blood for the 1 h and 6 h time points were collected from one of the subgroups, while blood for the 3 h and 12 h time points were collected from another. Blood and intestinal tract were also collected on day 7 after euthanasia. The small intestine consists of duodenum, jejunum and ileum and the proportion is 1:3:2. We divided the small intestine into 12 equal length segments (Intestinal Segment Number: ISN, ISN 1–12), and the cecum was one segment (ISN 13); the colon, consisting of proximal and distal parts, was separated into four equal length segments (ISN 14–17). Segments were numbered 1–17 from the most proximal to the most distal segment (Additional file 2: Figure S1). Segments were cut along the center axis and bent nose forceps were used to gently scrape the luminal contents, including undigested residue and intestinal mucosa, into 200 μL PBS buffer per segment.

Blood from mice that were intervened with deuterium-labeled isotopes was taken after the mice were fed for a week.

### Lipid extraction and derivatization

Total FAs were analyzed in food, blood, and gut tissues of experimental mice. Samples (20 μL or 20 mg) were extracted using the method of Bligh and Dyer [11], and a known amount of pentadecanoic acid (Sigma-Aldrich) and heneicosanoic acid (Sigma-Aldrich) was added as internal standard. After the extraction, the organic layers were combined and evaporated under a stream of nitrogen at 30 °C then dissolved in 1 mL of ethanol. Sample preparation was based on the method of Metcalfe et al. [12] as described previously [13]. Samples in ethanol were treated with 0.1 mL of 50% aqueous KOH, tubes were purged with nitrogen, and then heated to 75 °C for 1 h. After cooling, nonsaponifiable lipids were removed by extraction with 3 mL of hexane. The aqueous layer was acidified and extracted with 3 mL of hexane, and the hexane layer was removed and dried under a stream of nitrogen. The residue was treated with 0.5 M methanolic NaOH at 100 °C for 5 min. After cooling, samples were treated with 1 mL of 14% BF_3_ in methanol (Sigma-Aldrich), flushed with Ar, and then heated to 100 °C for 5 min. After cooling, samples were treated with 4 mL of hexane and 4 mL of saturated, aqueous NaCl, and the hexane layer was transferred to a new tube, dried under a stream of nitrogen, then dissolved with hexane (200 μL for blood samples or 1000 μL for tissue samples).

### GC/MS analysis

Fatty acids analyses were performed on GCMS-QP2010 Ultra (Shimadzu Co., Tokyo, Japan) with a Rtx-Wax column (0.25 mm × 30 m, 0.25 μm) (Restek International, Bellefonte, PA, USA). The column oven was initially kept at 40 °C for 5 min, and then the temperature was raised to 120 °C and 190 °C sequentially at a rate of 20 °C/min and 5 °C/min respectively. Oven temperature was kept at 190 °C for 5 min and then ramped to 220 °C at a rate of 5 °C/min and kept for 17 min. Helium was used as the carrier gas with a constant linear velocity of 0.94 mL/min. Temperature of GC inject, EI ion source and the interface were set at 240 °C, 220 °C and 250 °C, respectively. Full scan of *m/z* 50-550 was started at 3.0 min. Sample injection volume was 1.0 μL and the split ratio was 10.

### Statistics and multivariate data analysis

One-way Analysis of Variance (ANOVA) (SPSS 20.0, Chicago, IL, USA) was conducted to detect any differences between groups (Ctrl, n-3 PUFA and n-6 PUFA diet). Those variables (FA) with a p -value less than 0.05 were considered significant.

Multivariate data analysis was conducted by SIMCA-P+ software (Ver. 11, Umetrics, Umeå, Sweden). PCA (principal component analysis) biplot which shows both the scores and loadings was used to demonstrate the relationship between samples and the FA species.

## Results

### PUFA composition in blood responded swiftly and efficiently to dietary fat intake

To address the animal response to high and low ratios of n-6/n-3 PUFAs in the diet, we fed mice with synthetic diets containing different fatty acid compositions. The diets, including control, high n-3 and high n-6 diets, were nutritionally balanced with equal caloric input and total fatty acid amounts. Experimental diets with different amount of n-3 and n-6 PUFAs were prepared based on the desired n-6/n-3 PUFA ratios and verified by GC/MS analysis (Additional file 1: Table S1). There was no significant difference in daily food intake among the groups.

**Fig. 1:**
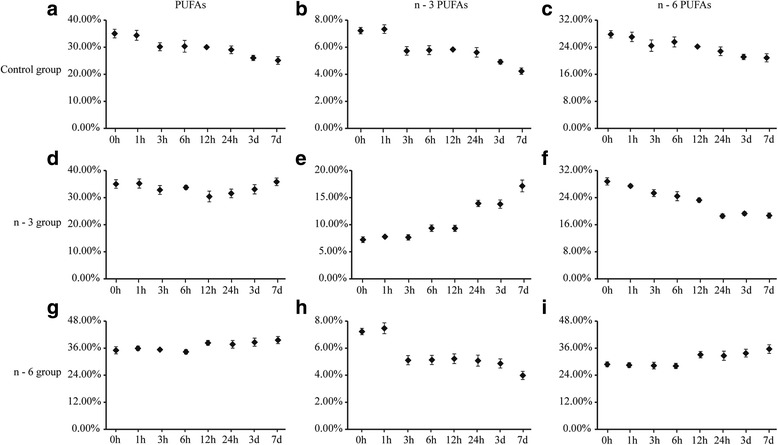
Composition of polyunsaturated fatty acids (PUFAs), n-3 PUFAs and n-6 PUFAs in blood. Polyunsaturated fatty acids (PUFAs), n-3 PUFAs and n-6 PUFAs are shown as a percentage of total FAs. **a**, **d**, **g** Blood PUFAs of the control, n-3 and n-6 PUFA groups. **b**, **e**, **h**: n-3 PUFAs of the three groups. **c**, **f**, **i**: n-6 PUFAs of the three groups

Fatty acid composition in the whole blood was analyzed at different time points after feeding mice with the experimental diets. As seen in Fig. 1, PUFA composition in mice whole blood, including n-3 PUFAs and n-6 PUFAs, was altered 3 h after dietary intake. After 7 days, the levels of n-3 PUFAs increased from 7.23 to 17.16% and the levels of n-6 PUFAs decreased from 28.81 to 18.69% in the n-3 diet group. When mice were fed with the n-6 diet, the levels of n-3 PUFAs in mice blood decreased from 7.23 to 3.99% and the levels of n-6 PUFAs increased from 28.81 to 35.59% (Fig. 1). Total levels of PUFAs, MUFAs and SFAs were similar to the fatty acid composition of the respective diets (Additional file 1: Table S1). These data clearly show that blood FA levels in mice responded to the high n-3 and n-6 PUFAs diets quickly and efficiently and reflected in part the fatty acid absorption in the intestinal tract. The blood lipid composition also reflected the dietary fatty acid content.

### Dietary intake of PUFAs modified the n-6/n-3 PUFA ratio and bioavailability

We measured the n-3 and n-6 PUFA in each segment, and the results suggest that the n-3 diet can significantly increase the bioavailability of n-3 PUFAs in the whole gut. In the n-3 group, the n-3 and n-6 PUFAs decreased along the GI tract from the anterior to posterior segments, dropping from 16.27 and 21.8 to 5.09 and 8.22%, respectively. Meanwhile, in the n-6 group, the n-3 and n-6 PUFAs decreased from 1.94 and 49.69% to 0.49 and 24.25%, respectively (Table 1). The n-6/n-3 PUFA ratios along the gut was altered correspondingly, ranging from 1.25 to 3.03 (n-3 diet) and 15.10-32.53 (n-6 diet) (Table 2). This indicates that, compared to its n-6 counterpart, n-3 PUFAs might have a more significant effect on the bioavailability of FAs along the gut as well as the n-6/n-3 PUFA ratio.

**Table 1:** n-3 polyunsaturated fatty acids (PUFAs) and n-6 PUFAs content (in% of total FA) of the 17 intestinal segments

ISN	Control group		n-3 group		n-6 group	
	n-3 PUFAs	n-6 PUFAs	n-3 PUFAs	n-6 PUFAs	n-3 PUFAs	n-6 PUFAs
1	2.68 ± 0.34	30.56 ± 0.84	16.27 ± 1.10	21.83 ± 1.83	1.94 ± 0.66	49.60 ± 1.14
2	2.42 ± 0.25	28.30 ± 1.86	15.84 ± 1.61	20.21 ± 1.51	1.38 ± 0.42	48.51 ± 6.60
3	1.90 ± 0.48	25.68 ± 7.88	14.06 ± 2.39	19.56 ± 1.73	1.17 ± 0.18	49.69 ± 2.82
4	2.38 ± 0.42	29.54 ± 5.54	15.82 ± 2.02	20.41 ± 2.91	1.13 ± 0.40	46.98 ± 5.08
5	2.09 ± 0.76	26.38 ± 7.62	15.67 ± 1.71	19.50 ± 2.32	1.00 ± 0.45	39.51 ± 5.79
6	2.77 ± 0.16	27.65 ± 7.21	13.24 ± 3.65	17.49 ± 2.28	1.53 ± 0.62	43.92 ± 9.39
7	1.76 ± 0.33	19.35 ± 1.95	13.52 ± 2.78	18.31 ± 1.76	1.79 ± 0.74	39.39 ± 5.11
8	2.14 ± 0.76	21.46 ± 5.63	6.10 ± 2.73	15.93 ± 2.53	1.92 ± 1.14	34.37 ± 8.04
9	2.79 ± 1.99	23.94 ± 10.2	5.90 ± 2.24	15.13 ± 2.37	3.06 ± 0.76	35.52 ± 5.08
10	1.76 ± 0.87	17.13 ± 5.65	12.80 ± 5.75	18.03 ± 2.52	1.98 ± 1.21	36.48 ± 7.75
11	2.81 ± 1.27	18.91 ± 5.84	13.13 ± 2.90	16.45 ± 1.99	0.81 ± 0.10	29.23 ± 3.69
12	1.14 ± 0.36	12.77 ± 2.28	9.38 ± 3.98	15.10 ± 2.99	0.67 ± 0.05	30.22 ± 4.86
13	1.98 ± 0.86	13.51 ± 2.33	5.62 ± 1.49	14.49 ± 3.80	1.31 ± 0.18	26.16 ± 1.50
14	1.76 ± 0.86	15.34 ± 2.18	5.76 ± 0.77	16.51 ± 3.77	0.49 ± 0.09	30.98 ± 1.97
15	2.25 ± 1.50	14.83 ± 4.98	5.09 ± 2.18	19.14 ± 3.44	1.17 ± 0.26	27.79 ± 2.63
16	2.83 ± 0.03	11.36 ± 1.44	7.56 ± 1.51	8.22 ± 2.36	0.71 ± 0.33	25.94 ± 2.35
17	2.34 ± 0.09	10.51 ± 2.77	5.38 ± 1.56	10.70 ± 4.96	0.81 ± 0.16	24.25 ± 2.29

Values are $\overline{x}\kern0.5em \pm \kern0.5em s$, as for n-3 PUFA3, there were no significance between Control and n-6 groups (*P* > 0.05 by ANOVA); and there were significant differences between n-3 and Control or n-6 groups (*P* < 0.05 by ANOVA) for n-3 PUFAs, as well as among Control, n-3 and n-6 groups (*P* < 0.05 by ANOVA) for n-6 PUFAs

**Table 2:** Ratios of n-6 polyunsaturated fatty acids (PUFAs) to n-3 PUFAs in the intestinal segments

ISN	Control group	n-3 group	n-6 group
1	11.67 ± 1.19	1.34 ± 0.07	29.07 ± 2.24
2	12.18 ± 1.25	1.28 ± 0.03	30.19 ± 2.55
3	12.66 ± 1.43	1.41 ± 0.20	32.53 ± 2.53
4	12.89 ± 0.53	1.30 ± 0.14	32.05 ± 1.80
5	12.46 ± 0.99	1.25 ± 0.09	31.48 ± 2.50
6	11.50 ± 1.46	1.35 ± 0.16	26.77 ± 2.82
7	11.48 ± 1.30	1.39 ± 0.14	22.83 ± 2.46
8	10.73 ± 1.42	2.70 ± 0.52	20.22 ± 1.69
9	11.51 ± 1.30	2.73 ± 0.30	15.10 ± 1.92
10	10.65 ± 1.25	1.73 ± 0.06	25.13 ± 1.65
11	9.97 ± 1.26	1.32 ± 0.26	26.72 ± 1.59
12	11.61 ± 1.11	1.83 ± 0.26	30.98 ± 1.24
13	10.35 ± 1.53	2.68 ± 0.25	26.64 ± 1.63
14	10.83 ± 1.82	2.88 ± 0.35	25.11 ± 1.96
15	11.35 ± 1.10	3.03 ± 0.34	22.07 ± 1.49
16	10.36 ± 0.47	2.23 ± 0.37	25.13 ± 1.43
17	11.90 ± 1.15	2.23 ± 0.09	31.13 ± 1.28

Values are $\overline{x}\kern0.5em \pm \kern0.5em s$, differences of ratios of n-6 PUFAs to n-3 PUFAs in all the intestinal segments were significant among Control, n-3 and n-6 groups (*P* < 0.05 by ANOVA)

The total content of PUFA, MUFA and SFA along the gut was determined and is shown in Table 3. We found that total PUFA levels decreased, while total MUFA levels increased along the intestinal tract in both n-3 and n-6 diet groups. However, the SFAs levels in the n-3 diet group were higher than in the other two groups, especially in the last two segments. This data hinted that the absorption of SFA was disturbed by the presence of n-3 PUFAs in the gut.

**Table 3:** Polyunsaturated fatty acid, monounsaturated fatty acid, saturated fatty acid content (in % of total FA) of the 17 intestinal segments

ISN	PUFAs (%)			MUFAs (%)			SFAs (%)		
	Control diet	n-3 diet	n-6 diet	Control diet	n-3 diet	n-6 diet	Control diet	n-3 diet	n-6 diet
1	33.2 ± 1.0	38.1 ± 2.8	51.5 ± 0.6	32.3 ± 2.4	23.2 ± 5.5	16.7 ± 2.0	31.9 ± 2.1	37.1 ± 4.6	29.8 ± 1.8
2	30.7 ± 1.7	36.1 ± 3.1	49.9 ± 6.5	35.4 ± 3.5	24.3 ± 6.8	19.3 ± 4.1	31.5 ± 3.2	37.9 ± 4.9	28.8 ± 3.5
3	27.6 ± 8.4	33.6 ± 3.8	50.9 ± 2.8	39.8 ± 8.4	29.5 ± 4.8	22.2 ± 5.2	30.2 ± 0.3	35.0 ± 2.2	25.3 ± 3.3
4	31.9 ± 5.9	36.2 ± 4.6	48.1 ± 5.4	32.3 ± 5.3	26.6 ± 5.4	22.9 ± 8.8	33.3 ± 2.9	35.2 ± 2.3	27.3 ± 4.1
5	28.5 ± 8.4	35.2 ± 3.9	40.5 ± 6.0	38.0 ± 10.6	26.1 ± 6.8	30.6 ± 6.3	30.5 ± 2.5	36.8 ± 3.8	27.0 ± 4.2
6	30.4 ± 7.3	30.7 ± 5.9	40.6 ± 3.2	32.9 ± 9.9	28.5 ± 7.9	20.4 ± 12.6	33.3 ± 3.2	38.8 ± 4.1	31.8 ± 8.2
7	21.1 ± 2.1	31.8 ± 4.5	41.2 ± 5.6	47.1 ± 5.3	26.7 ± 4.9	24.0 ± 6.9	29.3 ± 3.3	39.4 ± 1.7	32.2 ± 6.0
8	23.6 ± 6.2	22.0 ± 4.9	36.3 ± 9.1	41.8 ± 9.6	41.1 ± 8.5	25.2 ± 11.2	31.6 ± 2.8	35.2 ± 4.0	35.5 ± 1.6
9	26.7 ± 12.2	21.0 ± 3.4	38.6 ± 5.8	36.8 ± 18.6	42.0 ± 4.7	18.5 ± 5.6	33.1 ± 5.5	35.4 ± 2.0	38.4 ± 3.4
10	18.9 ± 6.5	30.8 ± 8.1	38.5 ± 8.1	51.0 ± 10.9	27.5 ± 11.7	25.7 ± 8.1	27.6 ± 5.5	39.0 ± 2.8	32.1 ± 5.6
11	21.7 ± 7.1	29.6 ± 4.0	30.0 ± 3.8	42.4 ± 4.5	24.7 ± 7.2	39.6 ± 3.9	32.4 ± 4.3	43.7 ± 4.6	28.2 ± 6.0
12	13.9 ± 2.6	24.5 ± 5.9	30.9 ± 4.8	55.5 ± 4.4	33.3 ± 8.9	36.9 ± 2.5	28.8 ± 3.0	40.7 ± 8.3	30.2 ± 3.8
13	15.5 ± 1.6	20.1 ± 2.8	27.5 ± 1.5	48.5 ± 7.1	44.1 ± 4.8	35.9 ± 3.0	32.9 ± 7.9	33.7 ± 7.1	33.6 ± 4.0
14	17.1 ± 2.0	22.3 ± 3.5	31.5 ± 2.0	41.0 ± 11.2	38.1 ± 5.5	40.3 ± 2.2	40.3 ± 13.4	38.2 ± 9.0	26.0 ± 2.5
15	16.5 ± 3.6	24.2 ± 1.7	29.0 ± 2.9	42.2 ± 12.7	42.9 ± 4.4	32.1 ± 1.6	38.8 ± 13.0	31.3 ± 5.7	35.5 ± 3.6
16	12.8 ± 2.7	15.8 ± 3.2	25.6 ± 1.2	35.3 ± 7.8	24.7 ± 6.7	32.9 ± 1.0	48.2 ± 10.0	57.4 ± 10.0	38.9 ± 2.3
17	11.7 ± 4.0	16.1 ± 5.0	24.8 ± 2.8	33.4 ± 4.9	30.8 ± 10.5	33.3 ± 3.2	50.7 ± 10.6	51.5 ± 15.5	39.5 ± 6.6

Values are $$ \overline{x} \pm \kern0.5em s $$

**Fig. 2:**
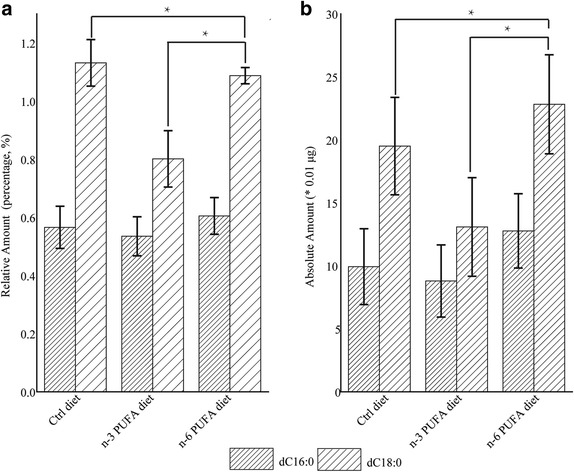
Accumulation of deuterium-labeled C16:0 (*d*_*31*_ C16:0) and C18:0 (*d*_35_ C18:0) in blood. Exogenous *d*_*31*_-C16:0 and *d*_*35*_-C18:0 in the blood on day 7 are shown in relative amount (as a percentage of the total blood fatty acids) (**a**) and in absolute amount (**b**) among control, n-3 and n-6 polyunsaturated fatty acid groups. One-way ANOVA analysis was performed by SPSS (IBM SPSS Statistics, Ver. 20) and those were of significance (*P* < 0.05) were marked with an asterisk (*N* = 5)

To further investigate this possibility, diets supplemented with deuterium-labeled FAs (*d*_31_-C16:0, *d*_35_-C18:0) were used to feed the mice. Results (Fig. 2) indicate that SFAs accumulation was comparable in the n-6 PUFA and control groups, and decreased in the n-3 PUFA group. One-way ANOVA analysis has shown that the change of *d*_35_-C18:0 were of significance (*P* < 0.05) between n-3 and control or n-6 groups. However, as for *d*_31_-C16:0, its change among the groups were of no significance. Possible reason for this might be that the exogenous *d*_31_-C16:0 were transformed to stearic acid [14] thus. This theory was validated by the facts that though almost equal amounts of *d*_31_-C16:0 and *d*_35_-C18:0 were added in the diets, the detected *d*_31_-C16:0 was half of that of *d*_35_-C18:0 (Fig. 2) and *d*_31_-C18:0 was detected from the mice blood (Additional file 3: Figure S2). These data suggest the dietary intake of high n-3 or n-6 PUFAs had different effects on the absorption of SFAs in the mouse gut.

### n-3 PUFAs influence the distribution of fatty acids in the gut

**Fig. 3:**
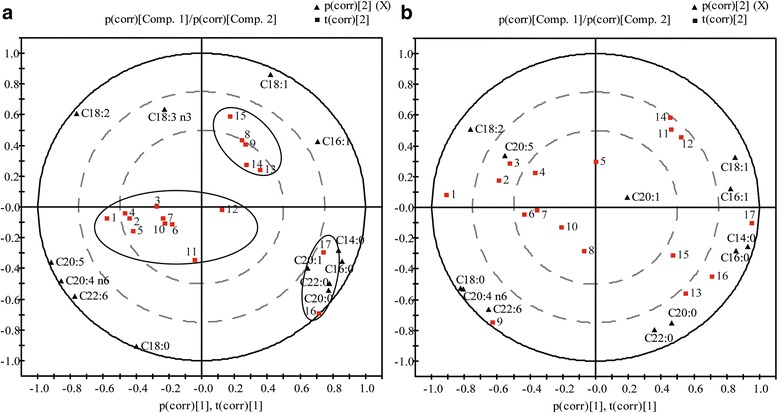
Principal component analysis (PCA) loadings biplot of intestinal fatty acids. **a** Biplot PCA of n-3 group, PC1 = 51.5%, PC2 = 31.7%; **b** biplot PCA of n-6 group, PC1 = 50.2%, PC2 = 23.7%. *Black triangles*: fatty acids; *red squares*: intestinal segments

A principal component analysis (PCA) was performed on intestinal FA data of n-3 and n-6 groups to further characterize the relationships between fatty acid species or classes and intestinal position or the intestinal environment, respectively. Segments which are clustered together have a similar fatty acid composition. In the loadings biplot (Fig. 3a and b), the n-3 group segments clustered well according to their position in the intestinal tract, whereas the n-6 group segments were scattered.

In n-3 group, the anterior end of the small intestine, including the duodenum, the major segments of the jejunum and ileum (ISN 1-7 and 10-12), with the exception of segments 8 and 9, were clustered as their PUFA composition consisted mainly of EPA (eicosapentaenoic acid, C20:5), DHA (docosahexaenoic acid, C22:6), LA (linoleic acid, C18:2) and AA (arachidonic acid, C20:4). And in more distal segments of the intestinal tract, the caecum and the proximal colon (ISN 13-15), the principal FAs were MUFAs (hexadecenoic acid, C16:1; octadecenoic acid, C18:1), whereas in the posterior end, mainly the distal colon (ISN 16-17), segments were clustered and closer to the SFAs (myristic acid, C14:0, palmitic acid, C16:0, eicosanoic acid C20:0, docosanoic acid, C22:0) (Fig. 3a and b). This could be a direct reflection of the absorption of different FAs along the gut, where dietary n-3 PUFA intake could induce differential absorption of PUFAs, MUFAs and SFAs. These data further indicate that dietary supplementation of n-3 PUFAs can enrich their bioavailability and modify the distribution of other fatty acids along the gut.

## Discussion

Epidemiological studies suggest that the consumption of n-PUFAs is beneficial for our health, and nutritionists often advise their patients to lower the n-6/n-3 fatty acid ratio in their diets [15]. To better understand the beneficial effects of a lower ratio of n-6/n-3 PUFAs, this study investigated the effects of different n-6/n-3 ratios on the organism by characterizing the FA composition along the intestinal tract and in the blood of C57BL/6J mice. Data showed that changing the n-6/n-3 PUFAs ratio in the diet could effectively alter n-6/n-3 PUFA ratios and the FA absorption along the gut.

Factors such as pH of the intestinal tract, bile acid secretion and microorganisms introduce drastic environmental change along the gut, and this might affect the absorption of dietary nutrients, including the FAs which are the focus of this work. As for fatty acids, there might be other factor such as fatty acid trasport proteins which effects their abosption. In a recent review of fatty acid transport proteins, Gomeno [16] stated that the new functions of these proteins are beening found, and they can also mediate the uptake of fatty acids. To interpret possible effects of n-3 and/or n-6 PUFA on the bioavailability and distribution of various FAs along the gut, mice were fed with experimental control, n-3 and n-6 PUFA diets for 7 days and were euthanized. The guts were taken and each was divided into 17 segments (Additional file 2: Figure S1) for fatty acid analysis. The quantity of total FAs in a given gut segment varied dramatically in individual mice, which may be related to the movement of the bowel along the gut. However, the percentage of each fatty acid was consistent. We therefore used the percentage to represent fatty acid content in each segment. Our data showed that the PUFA levels gradually decrease along the gut, from proximal to distal segments, which is consistent with the fact that the small intestine is the main organ where fatty acids are absorbed (Table 3). Through the progress of fatty acid absorption, the percentage of MUFAs along the gut became higher, while the SFA levels were steady, except in the last two segments. This phenomenon was particularly obvious when mice were fed with the high n-3 diet (Table 3). These data suggest that PUFAs could slow or prevent the absorption of SFAs, and n-3 PUFAs have a stronger effect than n-6 PUFAs.

Many factors could influence the absorption efficiency of fatty acids in the intestinal tract. Radiolabeled or stable isotopic fatty acid tracers, easily distinguishable from endogenic fatty acids, have be used to determine the absorption, digestion and interaction among the fatty acids in the neonatal piglets [17]. By feeding mice with diets mixed with deuterium-labeled SFAs, we have shown that the blood level of labeled SFAs declined significantly in n-3 diet group compared to the other two groups. These data are also consistent with the fact that SFAs were higher in the mouse gut in the n-3 diet group. These findings lead us to hypothesize that n-3 PUFAs can inhibit the absorption of saturated fatty acids in the mouse gut, through a mechanism that remains to be identified.

PCA biplots were constructed to visualize graphically the correlation between fatty acid species and intestinal position, as shown in Fig. 3. The results show that, after supplementation with n-3 PUFAs, segments along the whole gut were mainly clustered into 3 discrete sections, based on their enrichment in specific FA species: small intestinal segments (mainly PUFAs), caecum and proximal colon segments (mainly MUFAs) and distal colon segments (mainly SFAs). Unlike the majority of intestinal segments (1-7 and 10-12), intestinal segments 8 and 9 were closely clustered with segments of the caecum and proximal colon. It is not clear whether the very low total fatty acid contents in these two segments led to their deviation in the PCA biplot. Nonetheless, the results overall suggest that the uptake of fatty acid in the gut may occur in the order of PUFAs, MUFAs and SFAs, and n-3 PUFAs diets might enhance the differences in absorption efficiency of these different FAs (Fig. 3a). In the n-3 PUFA group, even though the FA analysis in the GI tract demonstrated reduced absorption of SFAs, a resulting decline of SFA levels in the blood was not observed. To test the possibility that this might due to a compensation of the de novo synthesis of SFAs in the mice, diets containing deuterium-labeled palmitic acid and stearic acid were used to feed the mice. Results from the stable isotope experiments confirm a decline in the assimilation of exogenous *d*_31_-C16:0 and *d*_35_-C18:0 in the blood of mice from n-3 PUFA diet group.

Various effects of PUFA, either n-3 or n-6 PUFA have been investigated [4, 18]. Undurti et al [10] studied the effect of fish oil with a high content of n-3 PUFA on mouse gut microbiota, and found that the fish oil treatment could change the gut microbiota. They proposed that this might be the potential mechanism of how n-3 PUFAs exert their biological effects. However, the specific mechanism may differ in different cases. Raghu et al reported the inhibition of intestinal β-carotene uptake by eicosapentaenoic acid (EPA) in their recent work [19]. They studied the effect of docosahexaenoic acid (DHA) and EPA on the absorption of β-carotene in intestinal cells and animal models. Their results shown that by down regulation of the scavenger receptor class B, type 1 via PPARα dependent mechanism, EPA and PPARα agonist could inhibit the uptake of β-carotene in Caco-2/TC7 cells. However, for DHA, PPARγ or PPARδ agonists, there were no such effect. This work indicated that other lipids maybe effected by n-3 PUFA in a similar way. Besides, it also shown that different species of n-3 PUFA may have different effects.

In our study, we systematically investigated the influence of dietary n-3 and n-6 PUFA intake on the distribution and bioavailability of fatty acids along the whole mouse gut. This study was made possible by using nutritionally balanced experimental diets with equal total fatty acid inputs, since the concentration of fat in the diet may influence the absorption of fatty acids [20]. Our results show that n-3 and n-6 PUFA composition in mice blood responded efficiently to experimental diets as early as 3 h after feeding, and reflected the dietary fatty acid input accurately. Gut fatty acid analysis revealed that an n-3 PUFA-rich diet can decrease the ratio of n-6/n-3 PUFAs along the gut and increase the bioavailability of n-3 PUFAs in the whole gut. Furthermore, by feeding the mice with diets containing isotope-labeled SFAs (*d*31-C16:0, *d*35-C18:0), we found that n-3 PUFA supplementation can reduce the absorption of saturated fatty acids in the gut. To our knowledge, this is the first report of this phenomenon.

## Conclusion

In the present work, we analyzed the distribution of blood and intestinal fatty acids of mice that were fed with home-made high n-3 and high n-6 fatty acid diets. The results indicated that supplements of n-3 fatty acids could increase the bioavailability of n-3 PUFAs, thus changed the ratio of n-6/n-3 PUFAs and altered fatty acid distribution. Besides, in the n-3 diet group, the absorption of saturated fatty acids (SFAs) along the gut was found to be inhibited and confirmed by feeding the mice with a diet containing deuterium-labeled palmitic acid and stearic acid.

## Additional files

Additional file 1: Table S1. Diet Ingredients and their fatty acid composition determined by gas chromatography/mass spectrometry (% and ratios). **Table S2.** Dietary intake data of the mice. (DOC 70 kb)
Additional file 2: Figure S1. Diagram of the location of different intestinal segments along the mouse gut. (TIF 496 kb)
Additional file 3: Figure S2. Extracted ion chromatograms (upper) and mass spectra (lower) of *d*_*35*_-C18:0 (333) and *d*_*31*_-C18:0 (329). (TIF 521 kb)
