# Supplementary material for: Dietary intake of n-3 PUFAs modifies the absorption, distribution and bioavailability of fatty acids in the mouse gastrointestinal tract
Source: Lipids Health Dis. 2017 Jan 17;16:10. doi: 10.1186/s12944-016-0399-9 (PMC5240384; doi:10.1186/s12944-016-0399-9)
Supplement: Additional file 1: Table S1. — Diet Ingredients and their fatty acid composition determined by gas chromatography/mass spectrometry (% and ratios). Table S2. Dietary intake data of the mice. (DOC 70 kb) [file 12944_2016_399_MOESM1_ESM.doc]

**Table S1.** Diet Ingredients and their fatty acid composition determined by gas chromatography / mass spectrometry (% and ratios)

| Ingredient | Diet Group (g/100 g Dry Weight) | | | | | | | | |
| --- | --- | --- | --- | --- | --- | --- | --- | --- | --- |
|  | Experimental diet | | | | | Stable isotope validation diet | | | |
|  | n-3 diet | n-6 diet | | Ctrl diet | | n-3 diet | n-6 diet | | Ctrl diet |
| **Variable diet fat** | | | | | | | | | |
| Palm oil | 8 | 6.5 | | 6.5 | | 8 | 6.5 | | 6.5 |
| Safflower oil | 0 | 6.5 | | 0 | | 0 | 6.5 | | 0 |
| Olive oil | 3 | 0 | | 6.5 | | 3 | 0 | | 6.5 |
| DHA^1^ | 1 | 0 | | 0 | | 1 | 0 | | 0 |
| EPA^1^ | 1 | 0 | | 0 | | 1 | 0 | | 0 |
| *d*_31_-C16:0 | 0 | 0 | | 0 | | 0. 14 | 0.14 | | 0.14 |
| *d*_35_-C18:0 | 0 | 0 | | 0 | | 0.14 | 0.14 | | 0.14 |
| **Fixed diet ingredients** | | | | | | | | | |
| Wheat flour | 30 | | | β-Sitosterol | | | 0.015 | | |
| Dextrin | 10 | | | Mineral salt mix^2^ | | | 4.7 | | |
| Sucrose | 10 | | | Vitamin mix^3^ | | | 1 | | |
| Casein | 10 | | | Choline Bitartrate | | | 0.25 | | |
| Lactalbumin | 10 | | | Tenox 20A | | | 0.006 | | |
| Alphacel | 11 | | |  | | | | | |
| **Fatty acid composition (% and ratios) of Control, n-3 and n-6 diets** | | | | | | | | | |
| Fatty acid | | | Control diet | | n-3 diet | | | n-6 diet | |
| C14:0 | | | 1.63±0.02 | | 1.02±0.03 | | | 1.11±0.01 | |
| C16:0 | | | 24.04±0.23 | | 27.15±0.46 | | | 22.50±0.09 | |
| C16:1 | | | 0.56±0.01 | | 0.39±0.01 | | | 0.29±0.02 | |
| C18:0 | | | 4.96±0.03 | | 3.83±0.14 | | | 4.36±0.09 | |
| C18:1 | | | 55.58±0.21 | | 45.58±0.45 | | | 28.79±0.02 | |
| C18:2 | | | 11.89±0.02 | | 10.22±0.21 | | | 41.02±0.01 | |
| C18:3 n-3 | | | 0.48±0.01 | | 0.34±0.01 | | | 0.91±0.01 | |
| C18:4 n-3 | | | ND | | 0.18±0.01 | | | ND | |
| C20:0 | | | 0.36±0.01 | | 0.32±0.02 | | | 0.48±0.01 | |
| C20:1 | | | 0.18±0.02 | | 0.19±0.01 | | | 0.19±0.01 | |
| C20:4 n-6 | | | ND | | 0.31±0.02 | | | ND | |
| C20:4 n-3 | | | ND | | 0.20±0.02 | | | ND | |
| C20:5 | | | ND | | 4.85±0.17 | | | 0.09±0.02 | |
| C22:0 | | | 0.31±0.01 | | 0.18±0.01 | | | 0.26±0.02 | |
| C22:5 n-6 | | | ND | | 0.34±0.01 | | | ND | |
| C22:5 n-3 | | | ND | | 0.36±0.03 | | | ND | |
| C22:6 | | | ND | | 4.54±0.25 | | | ND | |
| polyunsaturated fatty acids (PUFAs) | | | 12.37 | | 21.34 | | | 42.03 | |
| monounsaturated fatty acids (MUFAs) | | | 56.33 | | 46.16 | | | 29.27 | |
| Saturated fatty acids (SFAs) | | | 31.30 | | 32.50 | | | 28.70 | |
| n-3 PUFAs | | | 0.48 | | 10.47 | | | 1.01 | |
| n-6 PUFAs | | | 11.89 | | 10.87 | | | 41.02 | |
| n-6/n-3 PUFA | | | 24.61 | | 1.04 | | | 40.66 | |

1. Omega-3 H^TM^ EPA: 70% EPA & 10% DHA; omega-3 H^TM^ DHA: 75% DHA & 7% EPA from Huatai Biopharm Inc. (<http://www.huatai-biopharm.com/>).

2. Mineral salt mix: mg/Kg of diet (from AIN-93M-MX; Harlan Teklad, #TD94049): calcium carbonate, 357.0; potassium phosphate (monobasic), 250.0; potassium citrate (monohydrate), 28.0; sodium chloride, 74.0; potassium sulfate, 46.6; magnesium oxide, 24.3; ferric citrate, 6.06; zinc carbonate, 1.65; manganous carbonate, 0.63; cupric carbonate, 0.31; potassium iodate, 0.01; sodium selenate, 0.0103; ammonium paramolybdate (tetrahydrate), 0.008; sodium meta-silicate (nonahydrate), 1.45; chromium potassium sulfate (dodecahydrate), 0.275; lithium chloride, 0.0174; boric acid, 0.0815; sodium fluoride, 0.0635; nickel carbonate hydroxide (tetrahydrate), 0.0318; ammonium meta-vanadate, 0.0066; sucrose (fine ground), 209.496.

3. Vitamin mix: mg/Kg of diet (from AIN-93-VX; Harlan Teklad, #TD 94047): niacin, 3.0; calcium pantothenate, 1.6; pyridoxine HCl, 0.7; thiamin HCl, 0.6; riboflavin, 0.6; folic acid, 0.2; biotin, 0.02; vitamin B12 (0.1% in mannitol), 2.5; vitamin E (DL-alpha tocopheryl acetate, 500 IU/g), 15.0; vitamin A palmitate (500,000 IU/g), 0.8; vitamin D3 (cholecalciferol, 500,000 IU/g), 0.2; Vitamin K1 (phylloquinone), 0.075; sucrose (fine ground), 974.705.

**Table S2.** Dietary intake data of the mice

| Group | Diet Group (g/100 g Dry Weight) | | | | | | |
| --- | --- | --- | --- | --- | --- | --- | --- |
|  | Day 1 | Day 2 | Day 3 | Day 4 | Day 5 | Day 6 | Day 7 |
| n-3 diet | 6.21 | 4.38 | 4.31 | 4.55 | 4.48 | 4.41 | 4.41 |
| n-6 diet | 5.39 | 4.11 | 3.95 | 4.06 | 3.71 | 3.77 | 3.88 |
| Ctrl diet | 6.25 | 4.85 | 4.50 | 4.72 | 4.62 | 4.51 | 4.64 |
