# Supplementary figures and images for: Dietary intake of n-3 PUFAs modifies the absorption, distribution and bioavailability of fatty acids in the mouse gastrointestinal tract
Source: Lipids Health Dis. 2017 Jan 17;16:10. doi: 10.1186/s12944-016-0399-9 (PMC5240384; doi:10.1186/s12944-016-0399-9)

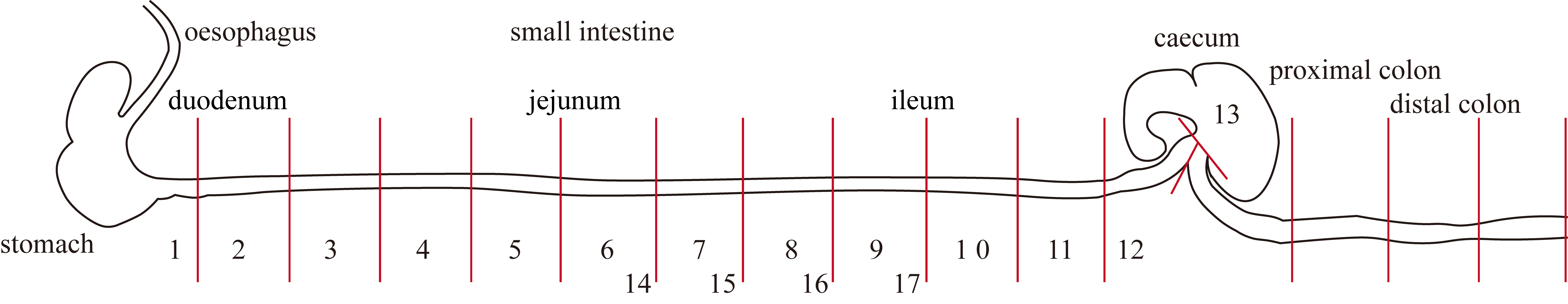

Supplement: Additional file 2: Figure S1. — Diagram of the location of different intestinal segments along the mouse gut. (TIF 496 kb) [file 12944_2016_399_MOESM2_ESM.tif]

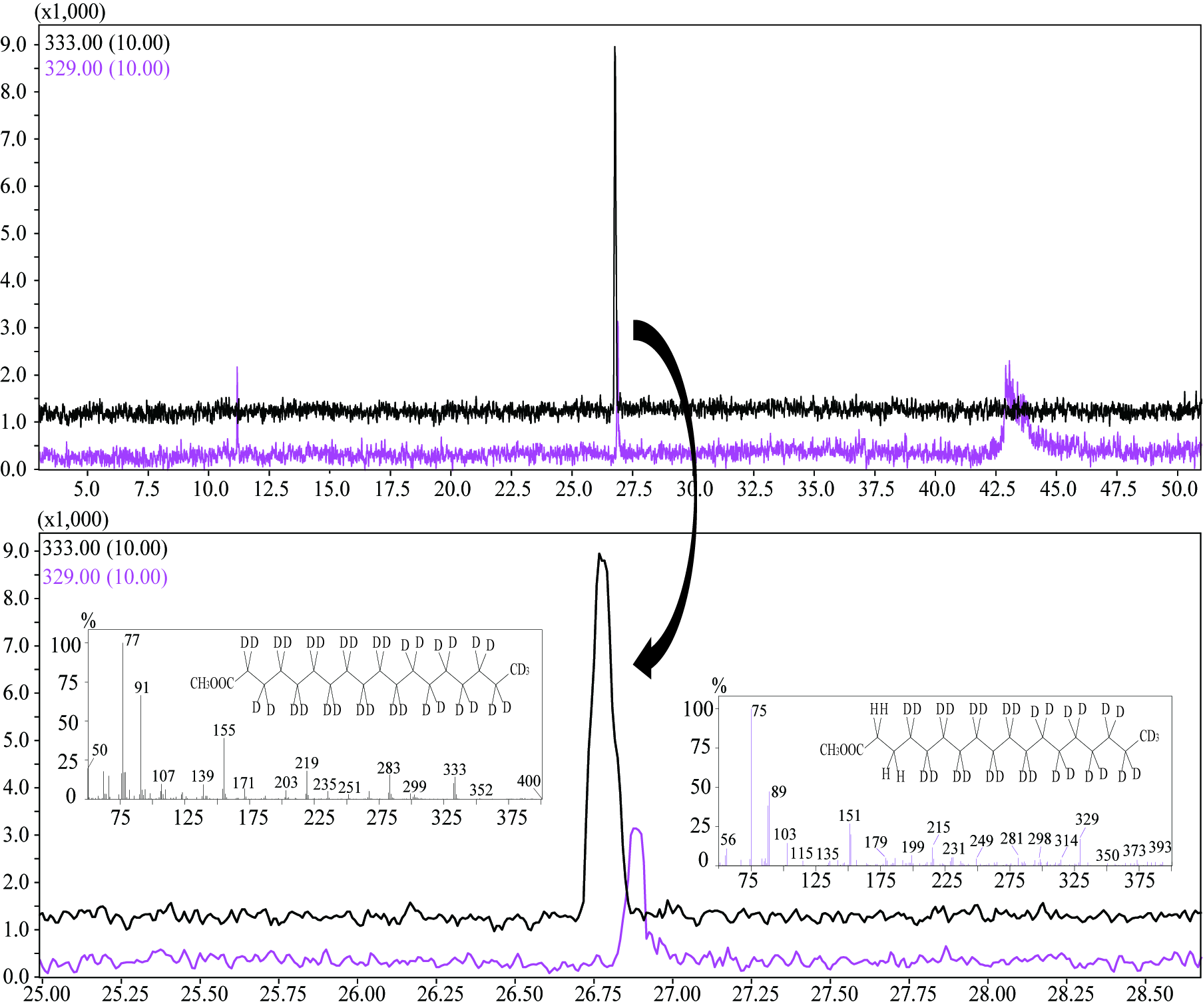

Supplement: Additional file 3: Figure S2. — Extracted ion chromatograms (upper) and mass spectra (lower) of d 35-C18:0 (333) and d 31-C18:0 (329). (TIF 521 kb) [file 12944_2016_399_MOESM3_ESM.tif]
